# Modelling staphylococcal pneumonia in a human 3D lung tissue model system delineates toxin-mediated pathology

**DOI:** 10.1242/dmm.021923

**Published:** 2015-11-01

**Authors:** Srikanth Mairpady Shambat, Puran Chen, Anh Thu Nguyen Hoang, Helena Bergsten, Francois Vandenesch, Nikolai Siemens, Gerard Lina, Ian R. Monk, Timothy J. Foster, Gayathri Arakere, Mattias Svensson, Anna Norrby-Teglund

**Affiliations:** 1 Department of Medicine Huddinge, Karolinska Institutet, Centre for Infectious Medicine, S-141 86 Stockholm, Sweden; 2 CIRI, International Center for Infectiology Research, Inserm, U1111, CNRS UMR5308, Université Lyon 1, École Normale Supérieure de Lyon, 69008 Lyon, France; 3 French National Reference Center for Staphylococci, Hospices Civils de Lyon, 69677 Bron Cedex, France; 4 Department of Microbiology, Moyne Institute of Preventive Medicine, Trinity College, Dublin 2, Ireland; 5 Society for Innovation and Development, Indian Institute of Science Campus, Bangalore 560012, India

**Keywords:** *Staphylococcus aureus*, Pneumonia, 3D lung tissue model

## Abstract

*Staphylococcus aureus* necrotizing pneumonia is recognized as a toxin-mediated disease, yet the tissue-destructive events remain elusive, partly as a result of lack of mechanistic studies in human lung tissue. In this study, a three-dimensional (3D) tissue model composed of human lung epithelial cells and fibroblasts was used to delineate the role of specific staphylococcal exotoxins in tissue pathology associated with severe pneumonia. To this end, the models were exposed to the mixture of exotoxins produced by *S. aureus* strains isolated from patients with varying severity of lung infection, namely necrotizing pneumonia or lung empyema, or to purified toxins. The necrotizing pneumonia strains secreted high levels of α-toxin and Panton-Valentine leukocidin (PVL), and triggered high cytotoxicity, inflammation, necrosis and loss of E-cadherin from the lung epithelium. In contrast, the lung empyema strain produced moderate levels of PVL, but negligible amounts of α-toxin, and triggered limited tissue damage. α-toxin had a direct damaging effect on the epithelium, as verified using toxin-deficient mutants and pure α-toxin. Moreover, PVL contributed to pathology through the lysis of neutrophils. A combination of α-toxin and PVL resulted in the most severe epithelial injury. In addition, toxin-induced release of pro-inflammatory mediators from lung tissue models resulted in enhanced neutrophil migration. Using a collection of 31 strains from patients with staphylococcal pneumonia revealed that strains producing high levels of α-toxin and PVL were cytotoxic and associated with fatal outcome. Also, the strains that produced the highest toxin levels induced significantly greater epithelial disruption. Of importance, toxin-mediated lung epithelium destruction could be inhibited by polyspecific intravenous immunoglobulin containing antibodies against α-toxin and PVL. This study introduces a novel model system for study of staphylococcal pneumonia in a human setting. The results reveal that the combination and levels of α-toxin and PVL correlate with tissue pathology and clinical outcome associated with pneumonia.

## Abstract (precis)

**Summary:** Use of a human 3D lung tissue model system identifies the combined effects of α-toxin and Panton-Valentine leukocidin in tissue injury and clinical outcome in staphylococcal pneumonia.

## INTRODUCTION

*Staphylococcus aureus* is an important cause of human infections, including respiratory tract infections. One of the most severe manifestations is community-acquired (CA) necrotizing pneumonia, which is associated with high mortality of 30-75% (Francis et al., 2005; Gillet et al., 2002). Reports have shown a strong epidemiological link between severe pneumonia and Panton-Valentine leukocidin (PVL)-positive CA *S. aureus* strains (Gillet et al., 2002, 2007).

Although some experimental studies have implicated PVL as a key contributor to necrotizing pneumonia (Diep et al., 2010; Gillet et al., 2002, 2007; Labandeira-Rey et al., 2007), others have implicated α-toxin, phenol-soluble modulins (PSMs) and surface protein A (BubeckWardenburg et al., 2007a,b, 2008; Olsen et al., 2010; Voyich et al., 2006). Thus, the defined role of the different toxins in the pathogenesis of necrotizing pneumonia remains unclear. Contradictory results can, at least in part, be explained by experimental systems using different hosts (rabbits and mice). Löffler et al. (2010) demonstrated that PVL induced rapid lysis of human and rabbit, but not murine or simian, neutrophils.

Further insight into host and cell specificity was provided by the identification of host receptors targeted by *S. aureus* pore-forming toxins (DuMont and Torres, 2014). The disintegrin and metalloprotease ADAM10, which is widely expressed on endothelial, epithelial and some immune cells, is the receptor for α-toxin (Wilke and BubeckWardenburg, 2010). The bi-component cytotoxins leukocidins LukAB, LukED and PVL target specific complement and chemokine receptors (Alonzo et al., 2013; Alonzo and Torres, 2013; DuMont et al., 2013), and the strict cell and host specificity of PVL could be linked to interspecies variation in C5aR (Spaan et al., 2013). This current knowledge underscores the importance of using a clinically relevant susceptible host for study of toxin-mediated pathology.

Most studies of human host-pathogen interactions are performed in two-dimensional (2D) cell culture systems, which poorly represent intact tissues. Alternatively, tissue explants are used, but have limitations on how they can be manipulated, particularly in humans. However, recent advances in creating robust and highly reproducible human three-dimensional (3D) tissue models (Nguyen Hoang et al., 2012), in which cellular constituents retain their differentiated phenotypes in an *in vivo*-like architecture, allow detailed study of tissue inflammation and infection under physiological conditions (Nguyen Hoang et al., 2014; Parasa et al., 2014).

This prompted us to establish an *in vitro* tissue model of *S. aureus* pneumonia based on human lung epithelial cells and lung fibroblasts. This 3D tissue model was employed to delineate the effects of specific *S. aureus* exotoxins in human lung epithelium as well as to test the efficacy of anti-toxin blocking therapy (i.e. polyspecific intravenous immunoglobulin G; IVIG). Collectively, the results revealed that the cytotoxicity mediated by α-toxin and PVL in combination resulted in the most severe tissue pathology. The toxin-mediated tissue damage was efficiently inhibited by IVIG. Thus, this novel *in vitro* model of pneumonia in a human tissue-like setting is a useful tool for mechanistic studies of disease pathogenesis as well as for testing novel therapeutic agents for pneumonia.

**TRANSLATIONAL IMPACT**

**Clinical issue**

Severe pneumonia caused by *Staphylococcus aureus* represents a major health problem. Several virulence factors, in particular the pore-forming cytotoxins α-toxin and Panton-Valentine leukocidin (PVL), have been implicated in the disease process but the mechanisms underlying the clinically relevant lung tissue destruction remains unclear. Many of the staphylococcal toxins exhibit a strong host- and cell-specificity, and human cells are particularly susceptible to them. Progress in the field has been hampered by the lack of experimental systems that allow for studies of toxin-mediated effects in human lung tissue. Here, the authors have used a novel approach including a human 3D lung tissue model to investigate the effect of specific exotoxins in human tissue and thereby gain an increased understanding of the pathogenic mechanisms leading to tissue injury in pneumonia.

**Results**

By exposing the 3D human lung tissue to specific *S. aureus* exotoxins produced by clinical pneumonia isolates, this study demonstrates that high levels of α-toxin directly damage the lung epithelium and that PVL contributes to tissue injury indirectly through the lysis of neutrophils. Furthermore, the study showed that the most severe tissue pathology is elicited by the combination of high concentrations of both α-toxin and PVL. Similarly, a collection of clinical *S. aureus* strains from individuals with pneumonia revealed that fatal outcome is linked to high toxin production and high cytotoxicity. In addition, α-toxin and PVL induced inflammation and strong upregulation of chemokines, subsequently causing increased neutrophil migration. Notably, both α-toxin- and PVL-mediated cytotoxic effects and tissue damage were completely abrogated by application of polyclonal intravenous immunoglobulin (IVIG) at physiological concentration.

**Implications and future directions**

This study demonstrates that the human 3D lung tissue model represents a useful tool for mechanistic studies of disease pathogenesis and for testing of novel therapeutic agents for pneumonia in a human-tissue-like setting. Furthermore, these findings identify dual actions of the toxins involving both cytolytic and chemotactic responses in the lung epithelium, demonstrating that toxin-mediated pathology is not limited to cytolytic events and underscoring the importance of targeting multiple toxins and inflammatory pathways in the treatment of severe *S. aureus* pneumonia. One therapeutic candidate is IVIG, which is polyspecific, targets multiple toxins and has immunomodulatory effects. This study shows that IVIG completely inhibits the toxin-mediated cytotoxicity and tissue injury and thus warrants further studies of IVIG as potential adjunctive therapy in *S. aureus* severe pneumonia.

## RESULTS

### Higher cytotoxicity and increased tissue pathology elicited by toxins secreted by necrotizing pneumonia isolates

**Fig. 1.**
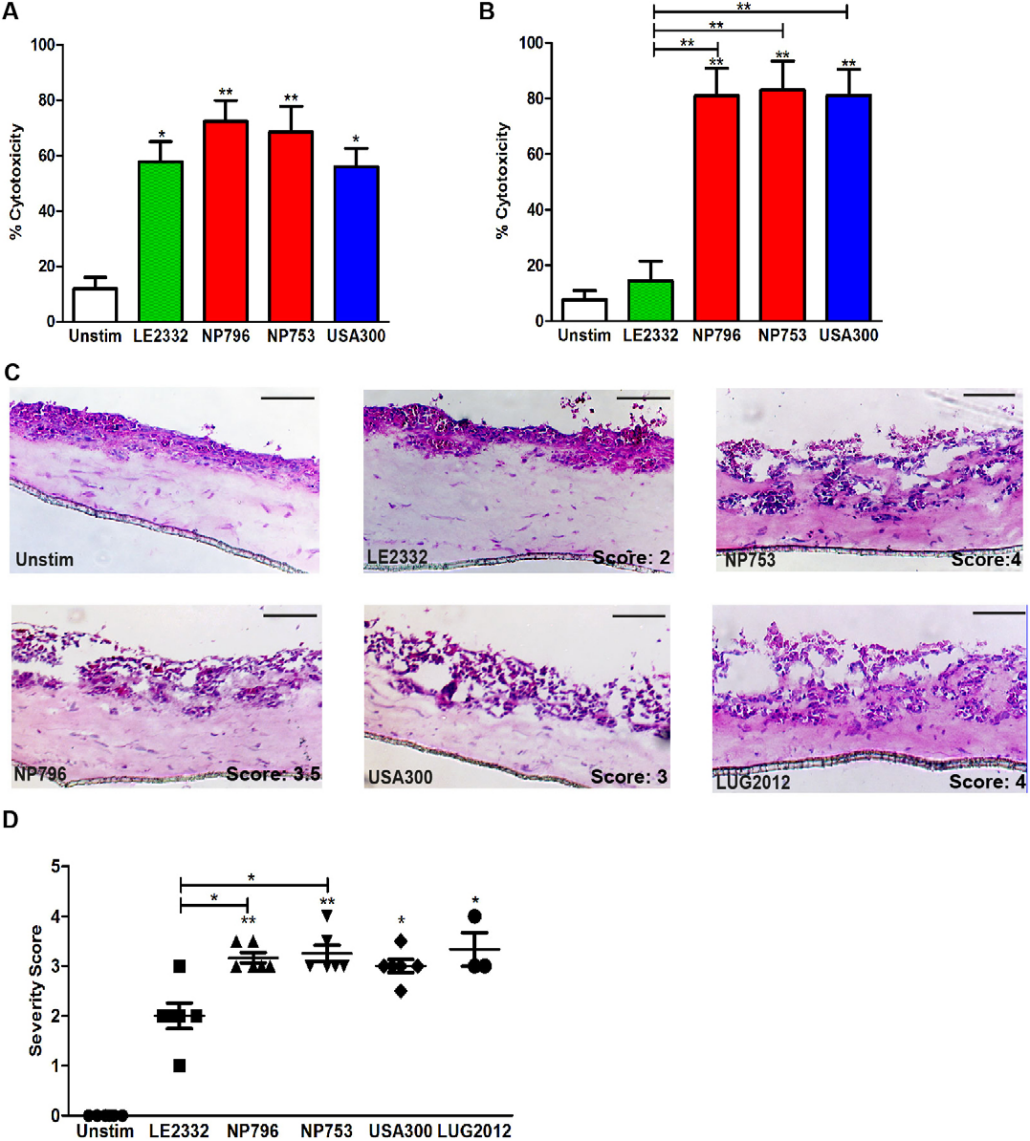
**Bacterial supernatant-mediated cytotoxicity and epithelial tissue injury.** (A,B) Cytotoxicity was determined by LDH measurements assessed in supernatants of neutrophils (A) and lung epithelial cells (16HBE) (B) exposed to bacterial supernatants prepared from cultures of LE2332 (green), NP753 (red), NP796 (red) and USA300 (11358) (blue). Results from unstimulated cells are shown in white. Neutrophils were exposed for 2 h and lung epithelial cells for 24 h, after which OD values were obtained. Percentage cytotoxicity is related to the positive control (Triton lysed cells). The bars show the mean±s.d. of five individual experiments. (C) Haematoxylin/eosin staining of cryosections of lung tissue models exposed for 24 h to bacterial culture supernatants of *S. aureus* NP796, NP753, LE2332, USA300 and LUG2012. (D) The scatter plot shows histological severity scoring data from three to six individual experiments. Scale bars: 100 µm. Statistically significant differences were determined by one-way ANOVA, Kruskal–Wallis, with Dunn's multiple comparison test; *P<0.05, **P<0.01 compared with unstimulated samples or comparison between samples as indicated by lines.

To study toxin-mediated tissue pathology that contributes to severe *S. aureus* pneumonia, we used clinical *S. aureus* strains isolated from patients with varying severity of pneumonia, including two severe cases of necrotizing pneumonia (strains NP796 and NP753) and one mild case of lung empyema (strain LE2332) (Table 1). Exotoxin-containing supernatants were prepared from overnight bacterial cultures and the toxin levels assessed (Table 1). First, human primary neutrophils and lung epithelial cells were exposed to the bacterial supernatants. All three strains were highly cytotoxic to human neutrophils (Fig. 1A). By contrast, only the culture supernatants from the necrotizing pneumonia strains elicited high cytotoxicity towards lung epithelial cells, including both bronchiolar (16HBE14o-) and alveolar (A549) epithelial cells (Fig. 1B, Fig. S1A). The USA300 CA-MRSA strain 11358 was used as a reference strain and was found to elicit a cytotoxic response similar to that of NP796 or NP753 (Fig. 1A,B, Fig. S1A).

**Table 1.**
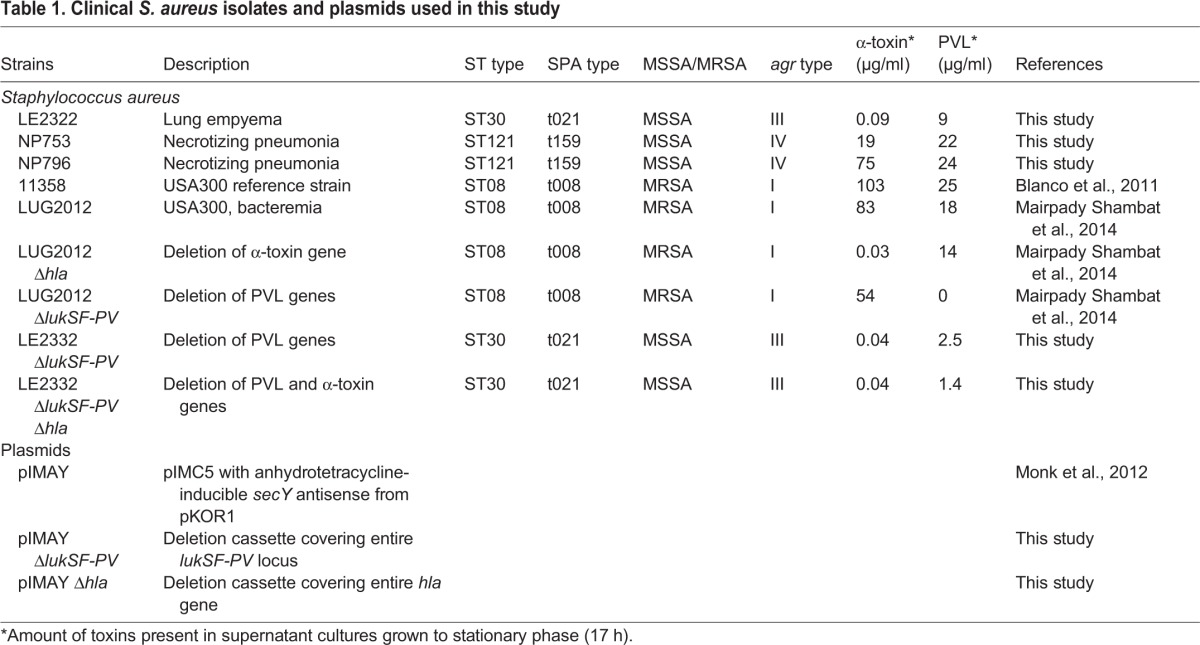
**Clinical *S. aureus* isolates and plasmids used in this study**

Next, we exploited 3D lung tissue models with an air-exposed stratified epithelial layer on top of a lung fibroblast matrix layer and studied the effect of bacterial supernatants. Histological analysis revealed that the lung tissue models responded in a highly reproducible manner to the different bacterial supernatants. The necrotizing pneumonia isolates, as compared with the lung empyema isolate, induced significantly greater disruption of the epithelial barriers and detachment of epithelial cells from the fibroblast matrix (*P*<0.05) (Fig. 1C,D). To ensure that the bacterial supernatant stimulations reflected a clinically relevant scenario, the lung tissue models were also infected with the necrotizing pneumonia and lung empyema strains and the tissue pathology assessed. All strains infected the tissue models, but the necrotizing pneumonia and USA300 strains demonstrated a greater dissemination than the lung empyema strain (Fig. S1C). At 24 h post-infection, the necrotizing pneumonia strains were found in deeper epithelial and fibroblast regions, were disseminated throughout the whole tissue model and induced severe injury and disruption of epithelium. Thus, the varying tissue pathologies elicited by the necrotizing pneumonia strains compared with the lung empyema strain were similar to those noted when bacterial supernatants were used.

### α-toxin directly disrupts the epithelium in lung tissue models

**Fig. 2.**
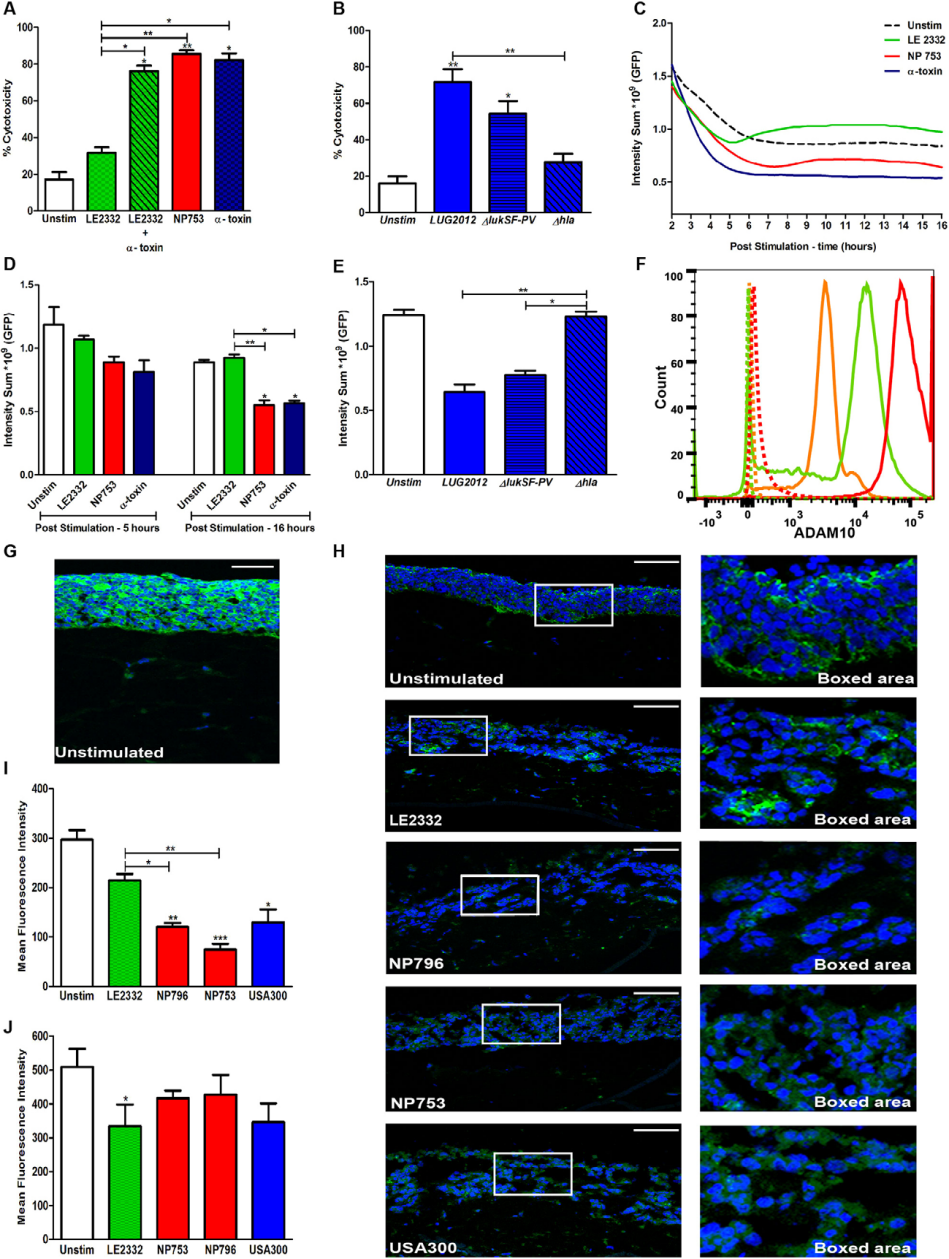
**α-toxin-mediated cytotoxicity and tissue injury.** Cytotoxicity elicited by bacterial supernatants and/or α-toxin were assessed in exposed cells and lung tissue models. (A) LDH release by human lung epithelial cells (16HBE) exposed for 24 h to culture supernatants of LE2332 and NP753, LE2332 supernatant supplemented with α-toxin (450 ng/ml), or α-toxin (450 ng/ml) alone. The bars show the mean±s.d. of three individual experiments. (B) LDH release by human lung epithelial cells (16HBE) exposed for 24 h to culture supernatants of LUG2012, LUG2012 Δ*lukSF-PV* and LUG2012 Δ*hla*. The bars show the mean±s.d. of three individual experiments. (C) Representative kinetic curves of intensity sum (GFP) acquired every 20 min for a period of 16 h post stimulation. The graph shows data from one representative experiment out of four individual experiments. (D) Intensity sum (GFP) of tissue models exposed to bacterial culture supernatants for 5 and 16 h. The bars show the mean±s.d. of four individual experiments. (E) Intensity sum (GFP) in lung tissue models exposed for 16 h to culture supernatants from LUG2012, LUG2012 Δ*lukSF-PV* and LUG2012 Δ*hla*. The bars show mean±s.d. of three individual experiments. (F) Flow cytometry data of ADAM10 expression in lung epithelial cells (red), lung fibroblasts (green), and neutrophils (orange). The graph shows data from one representative out of three individual experiments. (G) Immunofluorescence staining of ADAM10 (green) and cell nuclei (blue) in unexposed lung tissue models. (H) Immunofluorescence staining of E-cadherin (green) and cell nuclei (blue, DAPI) in lung tissue exposed to culture supernatants of strains LE2332, NP796, NP753, or USA300 (11358). Whole slide views (left panel) and magnified views of the boxed areas (right panel) are shown. The image shows data from one representative experiment out of three individual experiments. The mean fluorescence intensity was measured to determine the levels of E-cadherin expression (I) and claudin 1 (J). Scale bars: 100 µm. The mean±s.d. of three individual experiments is shown. Statistically significant differences were determined by one-way ANOVA, Kruskal–Wallis, with Dunn's multiple comparison test; *P<0.05, **P<0.01, ***P<0.001 compared with unstimulated samples or comparison between samples as indicated by lines.

To assess whether the differential cytotoxicity elicited by the bacterial supernatants was linked to particular toxins, the virulence gene profile was determined as well as quantification of certain toxins. The genes for α-toxin, PVL and PSMs were present in all three strains, but the two necrotizing pneumonia isolates differed from the lung empyema strain with respect to the genes encoding superantigens, LukED, proteases and staphylococcal superantigen-like proteins (Table S1) (Mairpady Shambat et al., 2014). However, quantification of the levels of α-toxin and PVL in the bacterial culture supernatants showed that the two necrotizing pneumonia isolates and the USA300 strains 11358 and LUG2012 all produced high levels of both α-toxin and PVL, whereas the lung empyema isolate produced moderate levels of PVL and negligible levels of α-toxin (Table 1). This suggested that α-toxin might be a key factor mediating the noted damage to the lung epithelium. To test this, we stimulated the lung epithelial cells with pure α-toxin at a concentration matching that present in the NP753 culture supernatant diluted 1:50, and measured the cytotoxicity. The results showed that α-toxin elicited a level of cytotoxicity equal to that induced by NP753, and that supplementation of LE2332 supernatant with α-toxin enhanced the cytotoxic response from around 30 to 75% (Fig. 2A). Also, the α-toxin-deficient mutant of LUG2012 lost its cytotoxic effect towards epithelial cells (16HBE and A549), whereas the PVL-deficient mutant had the same level of cytotoxicity as the wild-type strain (Fig. 2B, Fig. S1B).

Next, the kinetics of cytotoxic events was monitored by imaging green fluorescent protein (GFP)-expressing lung epithelium in real time. In this system, epithelial injury is associated with a reduction in GFP intensity (Fig. S2). Because of photobleaching in response to continuous laser exposure, the epithelium of all cultures displayed an initial reduction in GFP intensity, which stabilized in untreated tissue models (Fig. 2C). However, NP753 supernatant or pure α-toxin induced significantly more damage than did LE2332 supernatant (*P*<0.001) (Fig. 2D). Epithelial disruption was evident within 2 to 3 h and maximum damage was reached by 6 h (Fig. 2C). The culture supernatant of the LUG2012 α-toxin-deficient mutant displayed a cytotoxic profile similar to that of untreated tissue models, whereas the PVL-deficient mutant behaved similarly to the wild type (Fig. 2E). Taken together, the data indicate a direct damaging effect of α-toxin on lung epithelium.

To investigate whether differential expression of the α-toxin receptor ADAM10 could explain the varying susceptibility to α-toxin-mediated cytolysis, the surface expression of ADAM10 on lung epithelial cells, lung fibroblasts and neutrophils was analysed using flow cytometry. In agreement with the susceptibility of different cell types, the highest expression of ADAM10 was seen with lung epithelial cells, followed by fibroblasts, whereas neutrophils expressed the lowest levels (Fig. 2F). In lung tissue, ADAM10 was abundantly expressed and, in agreement with flow cytometry data, higher expression was noted in the stratified epithelial layer than in the fibroblast stromal layer (Fig. 2G).

Previous studies have shown that the interaction between α-toxin and ADAM10 results in activation of ADAM10 protease activity and, consequently, cleavage of E-cadherin (Inoshima et al., 2011). In line with this, analysis of E-cadherin in lung tissue models revealed a significant reduction in E-cadherin levels in models exposed to the culture supernatants of NP796 and NP753 as compared with LE2332 (Fig. 2H,I). This was not merely a consequence of loss of epithelial integrity, because expression of the tight junction protein claudin was only slightly reduced in models exposed to bacterial culture supernatants (Fig. 2J).

### PVL contributes indirectly to lung epithelial damage through neutrophil-mediated cytotoxicity

**Fig. 3.**
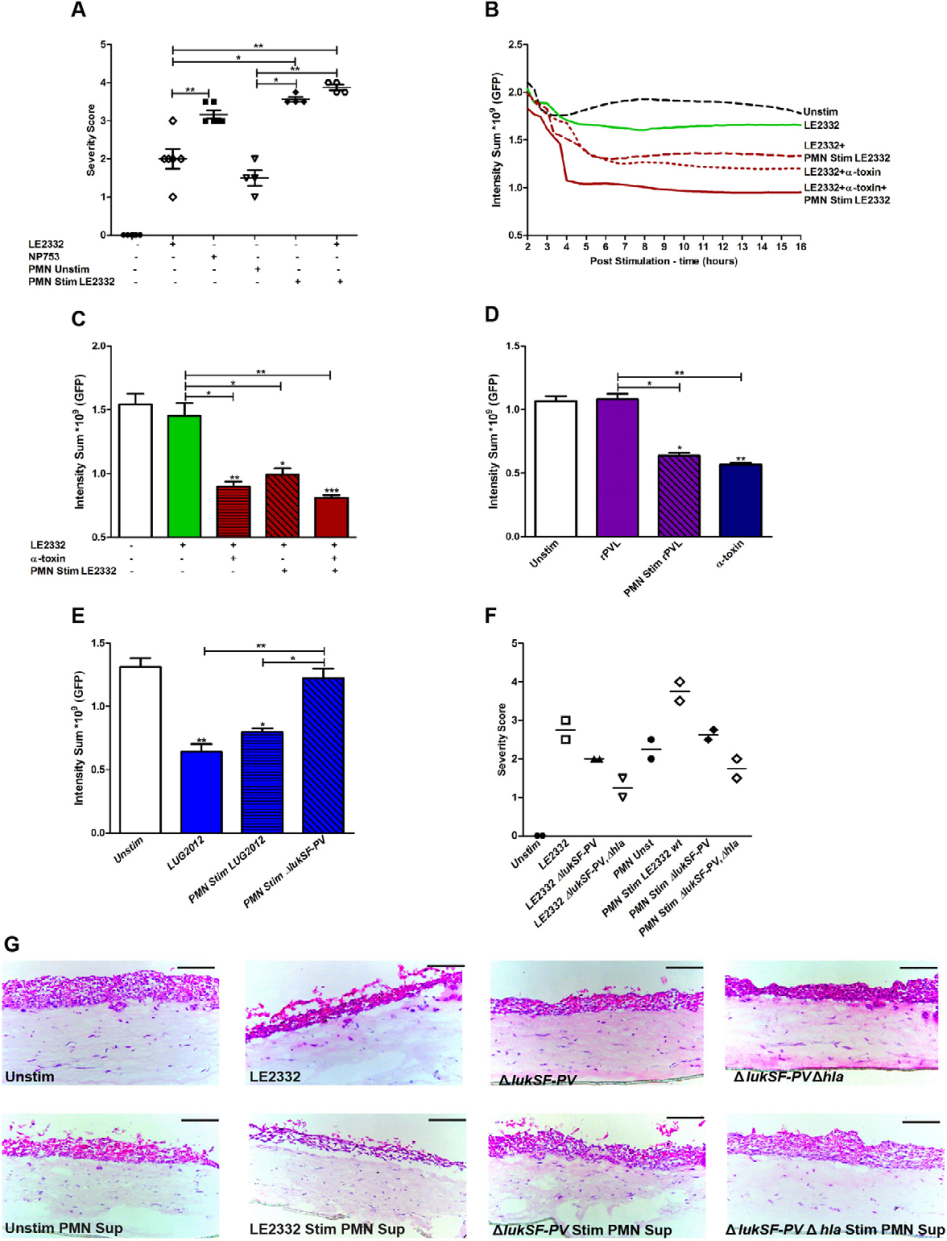
**PVL contributes to lung epithelial damage through neutrophil-mediated cytotoxicity.** (A) Histological severity scoring of lung tissue models exposed to culture supernatants from LE2332 or NP753, untreated neutrophils (PMN), and/or LE2332-exposed PMN. The scatter plot shows histological severity scoring data from four to six individual experiments. (B) Representative kinetic curves of intensity sum (GFP) acquired every 20 min for a period of 16 h post stimulation. The graph shows data from one representative out of three individual experiments. (C) Live image analysis of lung tissue models exposed for 16 h to bacterial culture supernatants from LE2332, pure α-toxin (450 ng/ml) and/or supernatant from LE2332**-**exposed PMN. The mean±s.d. of three individual experiments are shown. (D) Intensity sum (GFP) in lung tissue models exposed for 16 h to either rPVL (200 ng/ml), supernatants from neutrophils exposed to rPVL (200 ng/ml), or pure α-toxin (450 ng/ml). The mean±s.d. of three individual experiments are shown. (E) Intensity sum (GFP) in lung tissue models after 16 h exposure to culture supernatants from strain LUG2012 or to supernatants from PMN exposed to LUG2012 or LUG2012 Δ*lukSF-PV*. The mean±s.d. of three individual experiments are shown. (F) The scatter plot shows histological severity scores from two individual experiments. (G) Haematoxylin/eosin staining of cryosections of lung tissue models exposed for 24 h to (LE2332, LE2332 Δ*lukSF-PV*, LE2332 Δ*lukSF-PV* Δ*hla*) bacterial culture supernatants, supernatants from untreated PMN, and bacterial supernatant-exposed PMN. Scale bars: 100 µm. Statistically significant differences were determined by one-way ANOVA, Kruskal–Wallis, with Dunn's multiple comparison test; *P<0.05, **P<0.01 compared with unstimulated samples or comparison between samples as indicated by lines.

Next, we focused on cytotoxicity mediated by neutrophils and PVL. As shown in Fig. 1A, all bacterial supernatants, including that from the lung empyema strain, elicited a high level of cytotoxicity towards human neutrophils, consistent with the presence of moderate to high levels of PVL in the supernatants. To decipher the potential contribution of PVL-mediated lysis of neutrophils to tissue injury, attempts were made to embed the neutrophils into the lung model. However, the viability of primary neutrophils was not compatible with the long-term organotypic cultures. Instead, cell culture supernatants from neutrophils exposed to the bacterial supernatants were added to lung tissue models (Fig. 3A). The neutrophil culture supernatants were first characterized with respect to cytotoxic effects measured by LDH release. This revealed that LUG2012 and its α-toxin-deficient mutant elicited equally high levels of cytotoxicity, whereas the PVL-deficient mutant elicited reduced cytotoxicity (Fig. S3A). Similarly, LE2332 wild type elicited a high level of cytotoxicity, whereas its PVL-deficient mutant elicited an equally low level of cytotoxicity (Fig. S3B). Thus, the data suggested that PVL, but not α-toxin, elicited cytotoxicity towards neutrophils. This finding was further confirmed by use of pure α-toxin and recombinant PVL (rPVL) in different concentrations (Fig. S3C). Although the LE2332 culture supernatant triggered limited damage in the models (severity score 1-2), supernatants from LE2332-stimulated neutrophils resulted in epithelial destruction similar to that triggered by NP753 (severity score 3-3.5) (Fig. 3A). This was also evident in live imaging, where significant differences in GFP intensity were detected at 16 h (*P*<0.05) (Fig. 3B,C). The severity of LE2332-elicited tissue injury was significantly increased by addition of either pure α-toxin or supernatants from LE2332-stimulated neutrophils, with maximum damage noted when both α-toxin and supernatants from LE2332-stimulated neutrophils were present (Fig. 3B,C). Furthermore, the kinetics of these events revealed that maximum epithelial damage was evident earlier (3-4 h) in tissue exposed to both α-toxin and supernatants from LE2332-stimulated neutrophils as compared with other stimuli (Fig. 3B). Recombinant PVL alone did not elicit any direct cytotoxicity, but models stimulated with supernatants from PVL-treated neutrophils showed severe damage to epithelial cells, similar to that seen using pure α-toxin (Fig. 3D). We also exposed neutrophils to supernatants from toxin-deficient mutants of LUG2012 and LE2332. Severity scoring and live imaging analyses of exposed lung tissue models showed that loss of PVL, but not α-toxin, resulted in diminished cytotoxicity towards neutrophils and subsequent reduction in neutrophil-mediated epithelial damage (Fig. 3E-G). Taken together, this shows that both α-toxin and PVL contribute to lung epithelial injury by targeting different host cells.

### *S. aureus* toxins induce tissue necrosis, increased inflammation and chemokine responses in lung tissue models

**Fig. 4.**
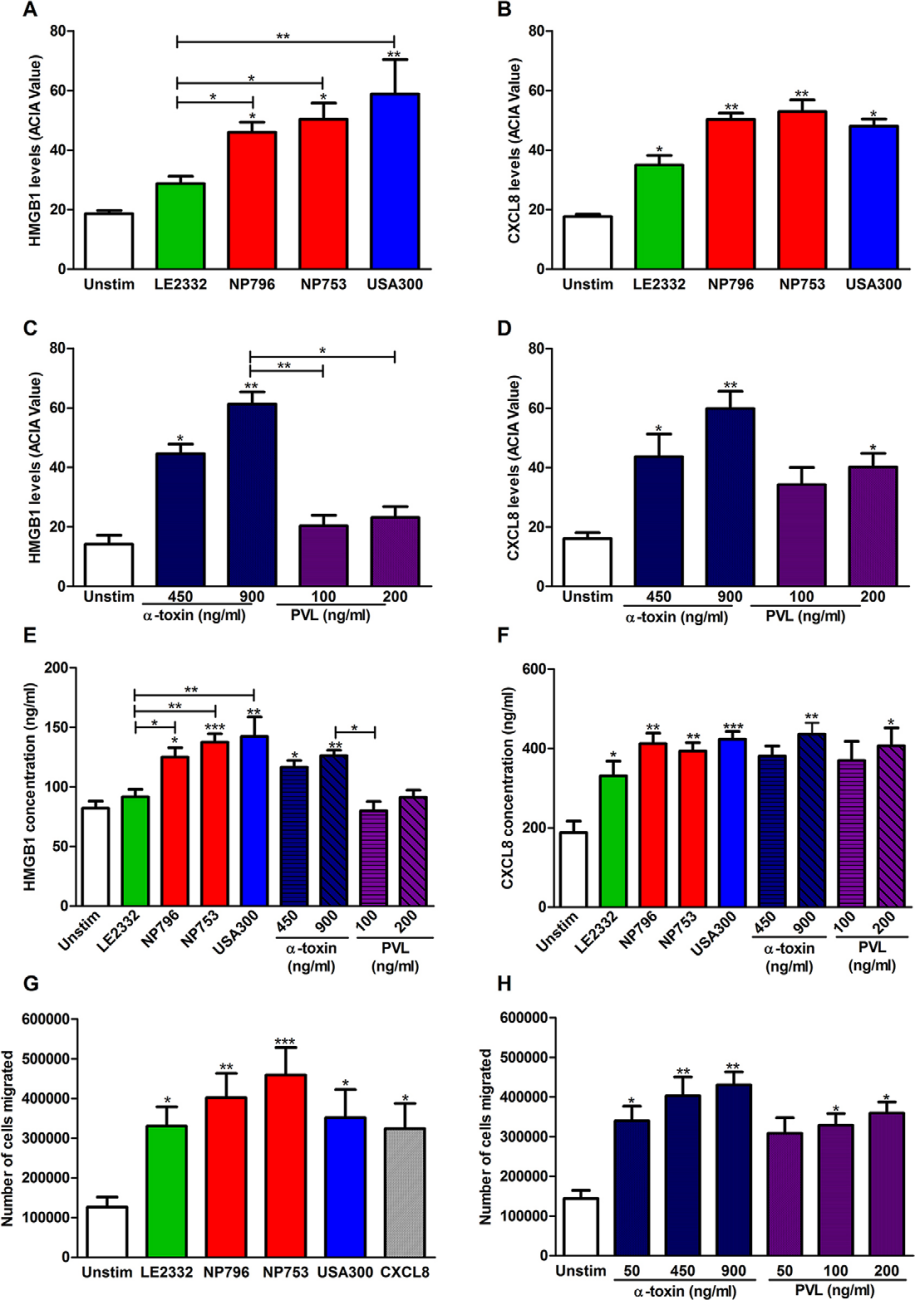
**Inflammatory and chemokine responses triggered by staphylococcal culture supernatants and pure toxins.** Lung tissue models exposed to culture supernatants from NP796, NP753, LE2332, USA300 or pure toxins were sectioned and immunohistochemically stained for HMGB1 and CXCL8. (A,B) Acquired computerized image analysis (ACIA) was used to obtain semi-quantitative levels of HMGB1 (A) and CXCL8 (B); mean±s.d. of four individual experiments are shown. (C,D) ACIA values for HMGB1 (C) and CXCL8 (D) in lung tissue models exposed to different concentrations of pure α-toxin (450 or 900 ng/ml) and rPVL (100 or 200 ng/ml). The mean±s.d. of three individual experiments are shown. (E,F) ELISA determination of HMGB1 (E) and CXCL8 (F) levels in supernatants of lung tissue models harvested after 24 h of exposure to culture supernatants of indicated bacterial strains or pure toxins. The mean±s.d. of four to eight individual experiments are shown. (G) Migration of neutrophils in a Transwell assay in response to supernatants from lung tissue models exposed for 24 h to culture supernatants of strains LE2332, NP796, NP753, USA300 or a positive control CXCL8 (25 ng/ml). Mean±s.d. of three individual experiments are shown. (H) Migration of neutrophils in a Transwell assay in response to supernatants from lung tissue models exposed to α-toxin (50, 450 or 900 ng/ml), rPVL (50, 100 or 200 ng/ml). Mean±s.d. of two individual experiments are shown. Statistically significant differences were determined by one-way ANOVA, Kruskal–Wallis, with Dunn's multiple comparison test; *P<0.05, **P<0.01, ***P<0.001 compared with unstimulated samples or comparison between samples as indicated by lines.

Because necrotizing pneumonia is characterized by an influx of neutrophils to the site of infection, we assessed the chemotactic signals in lung tissue models treated with bacterial culture supernatants. We found significantly increased levels of the neutrophil chemotactic factor HMGB1 in models exposed to supernatants from necrotizing pneumonia strains compared with that from the lung empyema strain (Fig. 4A, Fig. S4A). Notably, tissue models exposed to culture supernatants from LE2332, which elicited relatively mild tissue damage, demonstrated a low HMGB1 response whereas significantly increased levels of CXCL8 were detected (Fig. 4A,B, Fig. S4). To test whether this noted chemokine response is attributed to the actions of α-toxin and/or PVL, tissue models exposed to pure toxins were assessed for HMGB1 and CXCL8. The results revealed that the tissue model exposed to α-toxin, but not PVL, demonstrated high HMGB1 levels (Fig. 4C), whereas increased CXCL8 was found in models exposed to either PVL or α-toxin (Fig. 4D). Also, levels of secreted HMGB1 and CXCL8 in the lung tissue model media mirrored the immunostaining results, with elevated HMGB1 seen in response to high α-toxin but not to PVL, whereas CXCL8 secretion was triggered by both toxins (Fig. 4E,F). Measurements of tumour necrosis factor (TNF) and interleukin (IL)-1β in the lung tissue model media confirmed that the necrotizing pneumonia isolates and α-toxin induced relatively high inflammatory responses (Fig. S5). Taken together, these results demonstrate a direct toxin-mediated release of cytokines and chemokines in lung epithelium, evident even in the absence of cytolysis.

To explore further how this influences neutrophil migration, we tested the culture media harvested from stimulated lung tissue models in a Transwell migration assay. As illustrated in Fig. 4G, media from *S. aureus*-exposed tissue models induced neutrophil migration that exceeded that of the positive control, and the highest responses were seen with bacterial supernatants from the necrotizing pneumonia isolates. Also, supernatants from both PVL- and α-toxin-treated lung tissue models stimulated a strong chemotactic response over a broad concentration range and to a greater extent than the positive control (Fig. 4H).

### α-toxin and PVL production by clinical *S. aureus* pneumonia isolates relate to cytotoxicity and clinical outcome

**Fig. 5.**
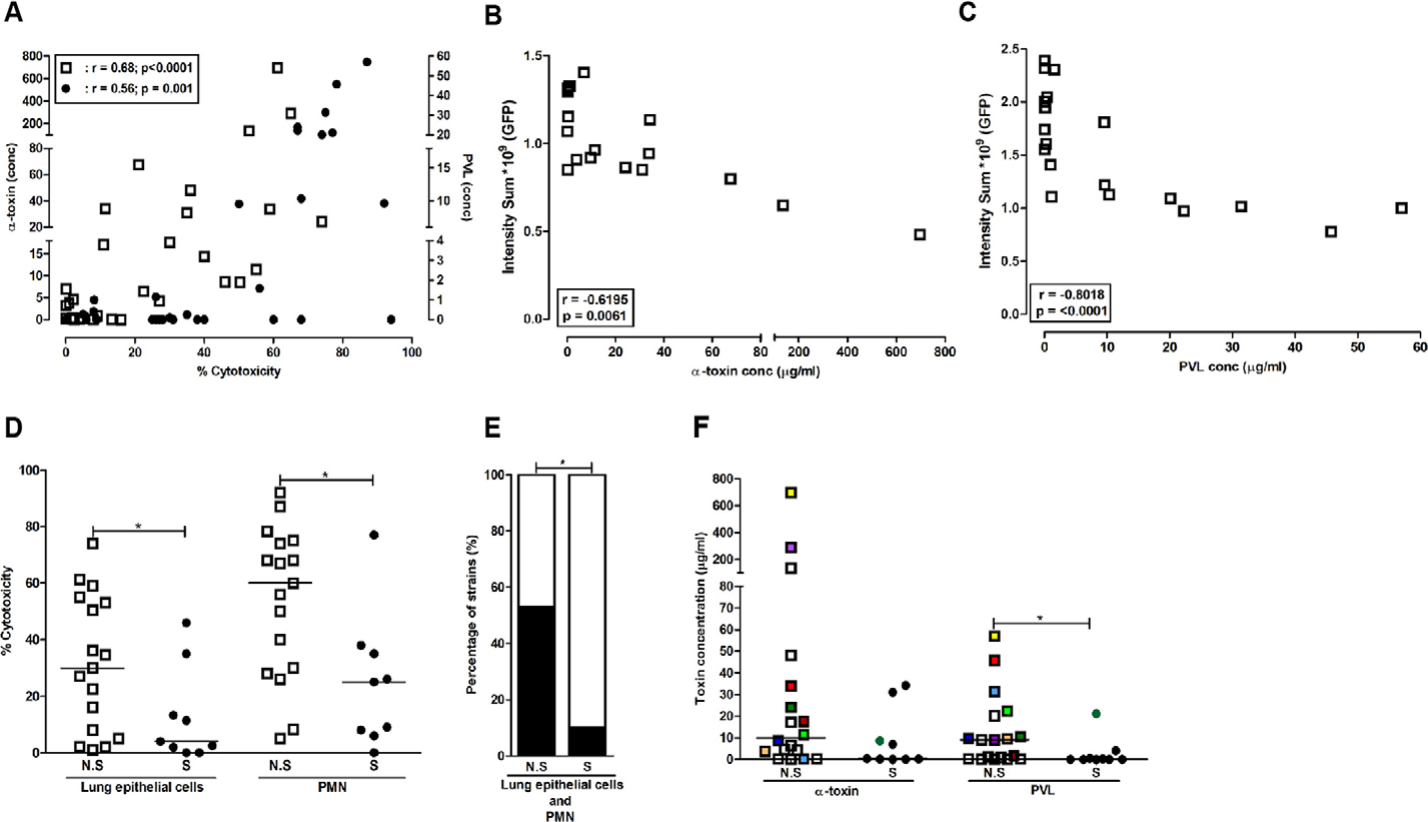
**Toxin levels and cytotoxic activity in supernatants from community acquired (CA) pneumonia isolates.** A collection of CA pneumonia isolates (*n*=31) was analysed for toxin levels and cytotoxic activity in bacterial culture supernatants. (A) Correlation of cytotoxic activity in bacterial culture supernatants towards lung epithelial cells versus α-toxin concentrations (left *y*-axis; open symbols) and neutrophils versus PVL levels (right *y*-axis; filled symbols). (B,C) Live imaging analysis of lung tissue models exposed for 16 h to culture supernatants from CA pneumonia strains (*n*=18, selected to obtain a representative collection of supernatants with varying α-toxin and PVL levels). The graphs show the correlation between epithelial injury (intensity sum) and levels of α-toxin (B) and PVL (C) in the culture supernatants. (D) Comparison between percentage cytotoxicity elicited towards lung epithelial cells and neutrophils (PMN) by strains isolated from non-survivors (NS) and survivors (S). (E) Strains were divided as eliciting high (>30%, black bars) or low (<30%, white bars) cytotoxicity. (F) α-toxin and PVL levels in bacterial supernatants of strains isolated from survivors (S) versus non-survivors (N.S). Pearson's correlation test was used in A-C, and *P* and *r* values are indicated. Statistical differences between N.S and S were determined using the Mann–Whitney test in D and F, and Fisher's exact test in E; *P<0.05.

Taken together, the data suggest that the most severe tissue pathology is elicited by the combined action of α-toxin and PVL. To test this in a clinical material, we analysed a collection of 31 CA pneumonia strains (Table S2) for toxin production and cytotoxicity. A positive correlation between α-toxin levels and cytotoxicity towards lung epithelial cells (*P*<0.0001) was noted, whereas PVL levels correlated with cytotoxicity towards neutrophils (*P*<0.001) (Fig. 5A). In exposed lung tissue models, injury was significantly correlated with levels of α-toxin present in the bacterial supernatants (*P*<0.006) (Fig. 5B). Similarly, neutrophil-mediated epithelial damage correlated in particular with strains expressing high levels of PVL (*P*<0.001) (Fig. 5C). Dividing the strains according to clinical outcome revealed that the strains from non-survivors elicited higher cytotoxicity against lung epithelial cells (*P*<0.05) and neutrophils (*P*<0.05) than strains from survivors (Fig. 5D). High cytotoxicity towards both epithelial cells and neutrophils was significantly more prevalent (50% versus 10%) among strains associated with fatal outcome (*P*<0.001) (Fig. 5E). Analysis of α-toxin and PVL levels produced by strains from non-survivors and survivors showed significantly higher levels of PVL in strains from non-survivors and a tendency towards higher levels of α-toxin (Fig. 5F).

### Toxin-mediated epithelial injury is blocked by IVIG

**Fig. 6.**
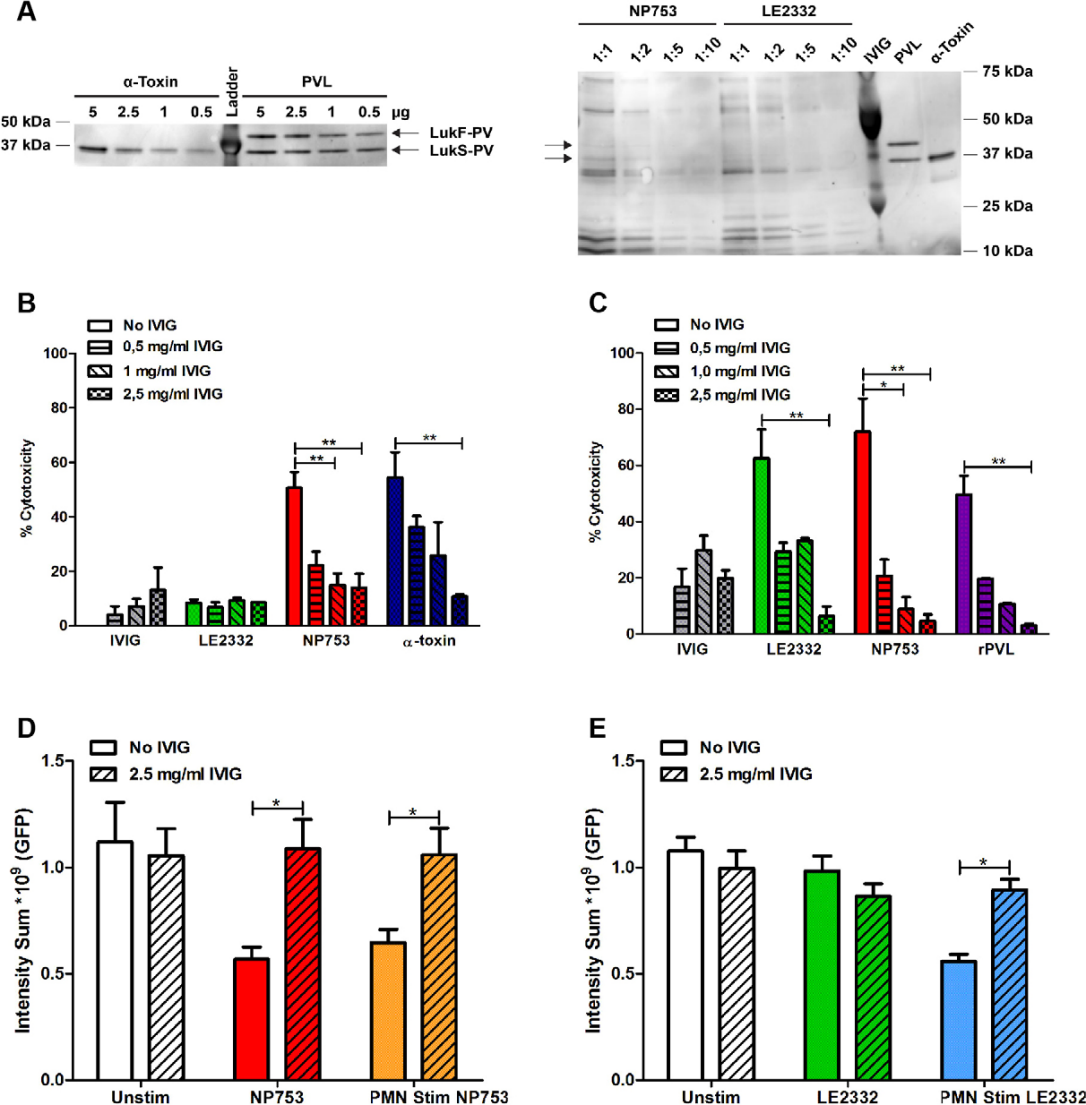
**α-toxin and PVL-mediated cytotoxicity is blocked by IVIG.** (A) Western blot analyses of α-toxin, rPVL and bacterial supernatants at indicated concentrations/dilutions. The blots were probed using IVIG as primary antibody followed by a secondary anti-human IgG antibody. (B,C) Lung epithelial cells (16HBE) (B) and primary human neutrophils (C) were exposed to bacterial supernatants from indicated strains or to toxins (α-toxin 500 ng/ml; rPVL 150 ng/ml) in the presence of different concentrations of IVIG. Cytotoxicity was assessed by lactate dehydrogenase (LDH) release assay. The bars show mean±s.d. of two individual experiments. (D) Intensity sum (GFP) in lung tissue models exposed for 16 h with 2.5 mg/ml IVIG alone, NP753, NP753+IVIG, supernatants from neutrophils (PMN) exposed to NP753, and supernatants from PMN stimulated with NP753+IVIG. Mean±s.d. of three individual experiments are shown. (E) Intensity sum (GFP) in lung tissue models exposed for 16 h to 2.5 mg/ml IVIG alone, LE2332, LE2332+IVIG, supernatants from PMN stimulated with LE2332, and supernatants from PMN exposed to LE2332+IVIG. Mean±s.d. of three individual experiments are shown. Statistical differences between presence or absence of IVIG were determined using the Mann–Whitney test; *P<0.05, **P<0.01.

IVIG has been reported to contain antibodies to both α-toxin and PVL and, thus, represents a potential adjunctive therapy in severe *S. aureus* pneumonia (Farag et al., 2013; Gauduchon et al., 2004; Rouzic et al., 2010). Here, we tested whether the noted pathology elicited by the toxins could be blocked by physiological concentrations of IVIG. The IVIG preparation used was found to contain antibodies to α-toxin, PVL and several factors present in the bacterial supernatants (Fig. 6A). A dose-response experiment was conducted in which lung epithelial cells and neutrophils were exposed to bacterial supernatants and α-toxin or PVL along with increasing concentration of IVIG. IVIG was found to efficiently block the cytotoxic effect of bacterial supernatants and pure toxins, with maximal effect at a concentration of 2.5 mg/ml (Fig. 6B,C). Live imaging of lung tissue models revealed that the epithelial damage mediated by the necrotizing pneumonia supernatant (Fig. 6D) or the neutrophils stimulated with bacterial supernatants (Fig. 6D,E) was completely abrogated by IVIG at physiological concentration.

## DISCUSSION

The human 3D lung tissue model system allows detailed study of toxin-mediated pathology in a tissue-like milieu and proved to be robust enough to delineate mechanisms of *S. aureus* pneumonia. The data obtained revealed that the combined action of α-toxin and PVL elicited the most severe epithelial injury, and this was further validated in a pneumonia strain collection. The finding that lung tissue models exposed to either α-toxin or PVL displayed a strong chemokine response, which resulted in increased neutrophil chemotaxis, demonstrates that toxin-mediated tissue pathology is not limited to cytolytic events.

Previous studies have explored the effect of α-toxin on different human lung epithelial cells, including 16HBE14o-, S9 and A549 cells, and shown that they are all sensitive to α-toxin-mediated cell death and respond with chemokine production (Hermann et al., 2015; Räth et al., 2013). However, the studies also revealed cell-specific differences with respect to the release of cytokines (i.e. IL-6) and the involved signalling mechanisms (Räth et al., 2013). In agreement with these studies, we also found 16HBE14o- and the alveolar cell line A549 to be equally susceptible to cytotoxicity elicited by staphylococcal supernatants and pure toxins. Therefore, in establishment of the 3D lung tissue model system, 16HBE14o- cells were used because they are non-cancerous and form proper epithelial barriers, whereas the adenocarcinoma A549 cells have functional tight-junction and epithelial barrier deficits (Heijink et al., 2010). Exposure of lung tissue models to toxins secreted by the clinical isolates resulted in varying responses and epithelial injury that correlated with their clinical severity. The necrotizing pneumonia isolates elicited significantly more severe tissue pathology than the lung empyema strain. Because the necrotizing pneumonia and lung empyema strains differed particularly with respect to their production of α-toxin, this toxin was implicated as a potential key mediator of the noted pathology. Further experiments using pure toxin and supernatants from toxin-deficient mutants verified that α-toxin elicited disruption of the lung epithelium, which is in line with previous findings (Bhakdi and Tranum-Jensen, 1991; Valeva et al., 1997; Vandenesch et al., 2012). Analyses of the α-toxin receptor, ADAM10, in the lung tissue model revealed that it was highly expressed and uniformly distributed across the epithelium, as in normal lung tissue. Also, a greater loss of E-cadherin was seen after exposure to supernatants containing high α-toxin levels. This is in line with the reported α-toxin-mediated activation of ADAM10 proteolytic activity resulting in E-cadherin cleavage (Inoshima et al., 2011; Wilke and Bubeck Wardenburg, 2010). Taken together, these data confirm previous reports of the effect and mechanisms of α-toxin on lung epithelial cells, including a direct cytolytic effect of α-toxin mediated by the proteolytic activity of ADAM10 on the adherens junction protein E-cadherin, probably contributing to disruption of the lung epithelium.

PVL elicited epithelial injury indirectly by triggering neutrophil lysis rather than via direct epithelial disruption. This is in agreement with previous studies demonstrating that epithelial damage is caused by granule proteases and other factors released during neutrophil lysis (Diep et al., 2010; Niemann et al., 2012; Van Wetering et al., 1997). Our data implicated PVL as the key mediator of neutrophil lysis because culture supernatants of PVL-deficient mutants had impaired lytic activity. Because fetal calf serum is used in our cell and tissue cultures and serum lipoproteins have been shown to inhibit PSMs (Surewaard et al., 2012), our experiments do not allow any conclusions to be made regarding the potential action mediated by these cytolytic peptides.

Our data further revealed that the combined action of α-toxin- and PVL-stimulated neutrophils resulted in maximum destruction of the lung epithelium. Also, analyses of a collection of clinical CA pneumonia strains of diverse lineages demonstrated that high toxin levels and cytotoxic activity in culture supernatants was associated with fatal outcome. In contrast, a previous study by Rose et al. (2015) demonstrated that nosocomial pneumonia strains showed an inverse relation between cytotoxicity and mortality. The discrepancy probably resulted from the fact that only neutrophil-like cells derived from the myeloid cell line HL-60 were used; thus, the assay only accounted for effects mediated predominantly by PVL and not α-toxin. Other differences include potential differences between cell lines and primary cells, and that hospital-associated MRSA isolates are more commonly associated with underlying conditions and are often less virulent than those of CA origin as recognized by several *in vivo* studies (Li et al., 2010; Voyich et al., 2005). Nevertheless, our data demonstrate that strains expressing high levels of α-toxin and PVL were predominantly found in the non-survivor cohort and provide clinical support for a combined role of high α-toxin and PVL in severe manifestations. This underscores the importance of assessing cytotoxicity in clinically relevant cell types as well as toxin levels, and not merely the presence of genes, in clinical and epidemiological studies.

Previous studies have suggested that *S. aureus*-triggered chemokine secretion in lung epithelial or innate immune cells contributes to the infection and severity of pneumonia (Bartlett et al., 2008; Escotte et al., 2006; Perret et al., 2012; Räth et al., 2013). Similarly, we found the inflammatory factors HMGB1 and CXCL8 significantly elevated and secreted when lung tissue models were exposed to supernatants from necrotizing pneumonia isolates compared with the lung empyema strain. Notably, these effects were seen even at sublytic concentrations of the toxins and subsequently increased secretion of chemotactic factors, resulting in enhanced neutrophil migration. Taken together, this effect of the toxins is likely to enhance neutrophil tissue infiltration and thereby exacerbate tissue pathology. This is similar to the model proposed for viral respiratory infection predisposing for *S. aureus* necrotizing pneumonia, in which the viral infection elicits increased chemokine expression and neutrophil influx (Niemann et al., 2012). Probably any inflammatory insult can serve as a predisposing event and, in terms of therapeutic interventions, these data underscore the importance of targeting multiple toxins and inflammatory pathways. Along these lines, we used IVIG as an anti-toxin blocking agent and found that it efficiently inhibited the lung epithelial injury elicited by α-toxin, PVL or bacterial supernatants in the 3D tissue models. This result supports the use of IVIG in pneumonia, which has been suggested in previous reports (Farag et al., 2013; Gauduchon et al., 2004; Rouzic et al., 2010).

In this study, we demonstrate that the 3D lung tissue model system provides a robust tool to model infections in a human tissue-like milieu in order to obtain a better understanding of *S. aureus* pneumonia pathology. Although the present 3D model system provides a relevant tissue-like environment with a complex cell composition, there are limitations, because real tissue has additional cellular constituents and is vascularized. In comparison with studies focusing on monolayers, the model provides an advantage as it allows study of the spatial distribution of host and bacterial factors, pathologic events in stratified epithelium, and relatively long-term infections. However, the model set up is laborious and time consuming, which is why monolayer cultures provide a good complement for high-throughput screening and optimization studies. Present work focuses on further optimization of the tissue model to include innate immune cells, such as myeloid cells, which will be central in future studies of tissue inflammation and infection control. Using these models in studies of physiological and pathological processes will help identify novel disease traits, and may point out new potential targets for the monitoring and treatment of human infectious diseases. In addition, the lung tissue model could be beneficial for pharmacological and toxicological studies, as well as for the evaluation of novel immunotherapeutic strategies.

## MATERIALS AND METHODS

### Bacterial strains

*S. aureus* isolates were collected from the pleural fluid of two necrotizing pneumonia cases (NP753 and NP796) and one lung empyema case (LE2332) (Table 1) at Global Hospitals, Hyderabad, India. The diagnosis of necrotizing pneumonia was confirmed by CT scan. The strains were characterized with respect to molecular typing including ST, *agr* types and toxin profiles (Table S1) (Mairpady Shambat et al., 2014; Shambat et al., 2012). USA300 (11358) was used as a reference strain (Blanco et al., 2011). The study also included 31 isolates from patients with CA *S. aureus* pneumonia from a French prospective cohort study (Gillet et al., 2002) and from cases referred to the French national reference laboratory for staphylococci (Table S2).

A clinical strain LUG2012 (USA300 lineage) and its isogenic mutants deficient in α-toxin (LUG2012 Δ*hla*) or PVL (LUG2012 Δ*lukSF-PV*) were used (Mairpady Shambat et al., 2014) (Table 1). Toxin-deficient mutants of strain LE2332 (Δluk*SF-PV* and Δ*lukSF-PV* Δ*hla*) were constructed by allelic exchange with pIMAY according to a previously described protocol (Monk et al., 2012). SLIC cloning of deletion constructs into pIMAY was conducted as previously described (Heilbronner et al., 2013). Deletion of the *hla* and *lukSF-PV* genes was validated by PCR and loss of toxin production tested by ELISA, as detailed below. Bacterial strains and plasmids used in this study are detailed in Table 1. Bacterial culture supernatants were prepared as previously detailed (Mairpady Shambat et al., 2014).

### Toxin ELISAs

Levels of α-toxin and PVL were determined by ELISA using toxin-specific antibodies provided by GSK Vaccines (Belgium) and bioMérieux R&D Immunodiagnostic (France), respectively (Badiou et al., 2010; Diep et al., 2013).

### Cell cultures and stimulation assays

Cells were cultured in complete RPMI 1640 medium containing 5% fetal calf serum, 2 mM L-glutamine, 100 U/ml penicillin, 100 µg/ml streptomycin and 1 M HEPES (all from Invitrogen). Epithelial cells were cultured in complete minimum essential medium (Sigma-Aldrich, St Louis, MO, USA) in fibronectin-coated plates.

Human neutrophils were isolated from blood obtained from healthy volunteers using Polymorphprep (Axis-Shield, Oslo, Norway) centrifugation. Blood was received from healthy volunteers. The study was done in accordance with the Helsinki declaration and approved by the ethical research committee at Huddinge University Hospital (Forskningskommitté Syd).

Neutrophils and lung epithelial cells, either bronchial 16HBE14o- (16HBE, a gift from Dieter Gruenert, Mt Zion, Cancer Center, San Francisco, CA, USA) or alveolar A549 (ATCC), were stimulated with bacterial culture supernatants (1:50 dilution) or different concentrations of pure α-toxin (Sigma-Aldrich) or recombinant PVL (IBT Bioservices, Gaithersburg, MD, USA) for 72, 2 and 24 h, respectively. Stimulated cells were analysed by flow cytometry and/or lactate-dehydrogenase (LDH) release assay as detailed below.

### Flow cytometry analysis

Stimulated cells were washed and incubated for 30 min on ice with conjugated antibodies: anti-CD3-FITC (clone SK7, R&D Systems), anti-CD45-Pacific Blue (clone T29/33, BD Biosciences) and anti-HLA-DR-Qdot 605 (clone TU36; Life Technologies) in combination with the dead cell marker Live/Dead Fixable near IR (Molecular Probes). Analyses were performed using a Beckton Dickinson LSRII SORP flow cytometer and FlowJo 9.5.3.

All cell types were stained with conjugated anti-ADAM10-PE (BioLegend, San Diego, CA, USA) and compared with their isotype control. Fluorescence was detected by flow cytometry analysis using same laser settings for all different cell types.

### LDH assay

Cytotoxic responses were measured by determination of LDH release into the tissue culture medium by cells that had been stimulated with bacterial culture supernatants or pure toxins. LDH was measured with a CytoTox 96 nonradioactive cytotoxicity assay kit (Promega) according to the manufacturer's protocol. The absorbance was read at 490 nm using a Microplate Manager 6 reader (Bio-Rad). Percentage cytotoxicity was determined in relation to the lysis control.

### 3D lung tissue model system

The epithelial cells, 16HBE14o- or A549 were used together with the human lung fibroblast cell line MRC-5 (ATCC) to set up the lung tissue models, essentially as previously described (Nguyen Hoang et al., 2012). Minor modifications included remodelling of the stroma/matrix layer for 7 days and use of 7×10^4^ epithelial cells. Lung tissue models were exposed on the apical side to bacterial culture supernatants diluted 1:50, different concentrations of pure toxins or stimulated neutrophil supernatants for defined time points, after which the tissue was frozen or used for live imaging experiments as detailed below.

### Histological analysis and immunostaining

For cryosectioning, lung tissue models were treated with 2.0 M sucrose for 1 h before embedding in optimum cutting temperature compound (Sakura Finetek), followed by freezing in liquid nitrogen and storage at −80°C. Cryosections of 8 µm were obtained using a MICROM cryostat HM 560 MV (Carl Zeiss) and fixed in 2% freshly prepared formaldehyde in PBS for 15 min at room temperature or in ice-cold acetone for 2 min at −20°C. For histological analysis, the sections were stained for 15 s in Mayer's haematoxylin and counterstained for 2 min in eosin. Histological severity scoring was performed in a double-blinded manner using the following criteria: 0 unaffected tissue, 1 mild injury with minor epithelial loosening, 2 moderate injury with some epithelial disruption, 3 severe injury with continuous epithelial disruption and some detachment, 4 extensive injury, massive epithelial disruption and detachment.

For immunohistochemistry, sections were stained as previously described (Johansson et al., 2014; Nguyen Hoang et al., 2012) using the following antibodies: polyclonal rabbit anti-HMGB1 (0.5 µg/ml; AbCam), mouse anti-CXCL8 (0.5 µg/ml, clone NAP-1; R&D systems), biotinylated goat anti-rabbit IgG (1:500) or goat anti-mouse IgG (1:600) (both from Dako). Immunostainings were visualized and quantified using a Leica DMR-X microscope and acquired computerized image analysis (ACIA) using the Qwin 550 software program (Leica Imaging Systems) as previously described in detail (Johansson et al., 2014; Nguyen Hoang et al., 2012; Parasa et al., 2014).

Immunofluorescence staining of sections was performed as previously described (Nguyen Hoang et al., 2012) using mouse anti-E-cadherin (2 µg/ml, clone HECD-1; Invitrogen), polyclonal rabbit anti-claudin1 (1 µg/ml; AbCam), mouse anti-ADAM10 (4 µg/ml, clone 11G2; Diaclone, Besançon, France), mouse anti-CXCL8 (4 µg/ml, clone NAP-1; R&D systems), and mouse anti-CX3CL1 (2 µg/ml, clone MM0207-8J23; Abcam). Specific staining was detected by Alexa Fluor 488-conjugated donkey anti-mouse IgG and Alexa Fluor 488-conjugated donkey anti-rabbit IgG (3.3 µg/ml) (both from Molecular Probes). Staining was visualized using a Nikon A1 confocal microscope (Nikon Instruments). The mean fluorescence intensity (MFI) in 10-15 fields per tissue section was determined using NIS element AR image analysis software (Nikon).

### Live imaging of 3D lung tissue models

For live imaging, lung tissue models constructed with GFP-expressing epithelial cells were used (Nguyen Hoang et al., 2014). The models were stimulated with bacterial culture supernatants or pure toxins as outlined above. The lung tissue models were incubated at 37°C for 1.5 h, mounted and analysed in real-time as previously described (Nguyen Hoang et al., 2014). Live imaging was performed for 16 h at 20 min intervals by acquiring 3D *z*-stacks with a 3 μm *z* resolution in an image volume of 512×512 μm in the *x* and *y* directions and 120 μm in the *z* direction with an 20× air objective (Plan Apo VC 20x DIC N2, numerical aperture 0.80) in 5% CO_2_ at 37°C using Nikon A1R spectral detector confocal microscopy (Nikon Instruments). The total intensity sum of GFP signal for each time point was determined using Imaris image analysis software (Bitplane).

### HMGB1 and CXCL8 ELISA

The levels of HMGB1, CXCL8, TNFα and IL-1β in supernatants from stimulated lung tissue models were measured using human CXCL8, TNFα and IL-1β Quantikine ELISA (R&D Systems) and human HMGB1 ELISA (IBL international) according to the manufacturers' instructions. All samples were analysed in duplicate.

### Transmigration chemotaxis assay

The chemotactic effect of supernatants collected from stimulated lung tissue models was assessed using a Transwell migration assay. The supernatants collected from stimulated lung tissue models (diluted 1:50 in RPMI 1640 complete media) were added to the outer chamber of Transwell plates (Costar, Corning Inc.). CXCL8 (25 and 100 ng/ml) was used as positive control. Neutrophils were seeded at 5×10^5^ cells/well in the upper chamber of a 24-well Transwell plate and incubated for 2 h at 37°C. To quantify cellular migration, cells were collected and mixed with a fixed amount of Count Bright absolute counting beads (Molecular Probes). Analysis was done using a BD LSRII Fortessa cell analyser (BD Bioscience) with gating on neutrophils and beads, respectively, to obtain the total number of cells that had migrated. FlowJo software version 9.5.3 (Tree Star) was used for flow cytometry analyses.

### Western blot analysis

Pure α-toxin or rPVL at concentrations ranging from 0.5 to 5 µg/ml, or serial dilutions of bacterial culture supernatants, were boiled for 5 min in sample loading buffer (Invitrogen). The samples were separated by 12% SDS-PAGE and transferred to a PVDF membrane. As molecular mass marker, pre-stained protein standards (Bio-Rad) were used. The membranes were blocked with 5% skim milk and incubated with primary antibodies (0.5 µg/ml IVIG), washed and then incubated with secondary antibodies (anti-human IgG horseradish peroxidase linked Fab fragment) (GE Healthcare). Positive binding was detected by Super Signal West Femto maximum sensitivity substrate (Thermo-Scientific, IL, USA).

### Statistical analysis

The data were analysed using the GraphPad Prism v.5 software (GraphPad, San Diego, CA). Statistically significant differences were determined by use of the Mann–Whitney test or the Kruskal–Wallis with Dunn's multiple comparison test. Correlations were determined by Pearson's correlation test. Differences were considered significant for *P*<0.05.
